# Feasibility of high intensity interval training in patients with breast Cancer undergoing anthracycline chemotherapy: a randomized pilot trial

**DOI:** 10.1186/s12885-019-5887-7

**Published:** 2019-07-03

**Authors:** Kyuwan Lee, Irene Kang, Wendy J. Mack, Joanne Mortimer, Fred Sattler, George Salem, Christina M. Dieli-Conwright

**Affiliations:** 1 Division of Biokinesiology and Physical Therapy, Ostrow School of Dentistry, University of Southern California (USC), 1540 E. Alcazar St., CHP 155, Los Angeles, CA 90089 USA; 2 Department of Medicine, Keck School of Medicine, University of Southern California (USC), Los Angeles, CA 90089 USA; 3 Department of Preventive Medicine, Keck School of Medicine, University of Southern California (USC), Los Angeles, CA 90089 USA; 4 Division of Medical Oncology & Experimental Therapeutics, City of Hope Comprehensive Cancer Center, Duarte, CA 91010 USA

**Keywords:** High intensity interval training, Peak power output, Anthracycline, Breast cancer, Feasibility

## Abstract

**Background:**

Anthracycline-based chemotherapy is associated with reduced cardiorespiratory fitness in breast cancer patients. High intensity interval training (HIIT) induces greater benefits on cardiorespiratory fitness than moderate continuous aerobic exercise in patients with heart failure. The study purpose was to determine whether a HIIT intervention is a feasible exercise strategy for breast cancer patients undergoing anthracycline-based chemotherapy.

**Methods:**

Thirty women were randomized to either HIIT or non-exercise control group (CON). Participants performed a maximal cycling fitness test to measure peak power output during maximal oxygen uptake (VO_2_max). The HIIT group participated in an 8-week HIIT intervention occurring 3 times weekly. Feasibility was calculated by computing (1) the average weekly minutes of HIIT over 8 weeks and (2) the number of sessions attended and multiplied by 100 (percentage of sessions). The intervention was considered feasible if more than 50% of participants completed both an average of 70% of weekly minutes (63/90 min) and attended 70% exercise sessions (17/24 sessions).

**Results:**

Participants were 46.9 ± 9.8 (mean ± SD) years old, diagnosed with clinical stage II (30%) or III (63%) breast cancer. The average weekly minutes of exercise completed was 78 ± 5.1 out of 90 min. Twelve of 15 participants met both feasibility criteria, attending 19.2 ± 2.1 out of 24 sessions (82.3%). VO_2_max was maintained (19.7 ± 8.7 to 19.4 ± 6.6 ml/kg/min) in HIIT group (*p* = 0.94) while there was a significant decrease in VO_2_max (18.7 ± 7.1 to 16.1 ± 6.0 ml/kg/min) in CON group from baseline to 8 weeks (*p* = 0.001).

**Conclusions:**

HIIT is a feasible exercise intervention to maintain VO_2_max in breast cancer patients receiving anthracycline-based chemotherapy.

**Trial registration:**

The protocol and informed consent were approved by the institutional IRB (HS-12-00227) and registered (ClinicalTrials.gov NCT02454777; date of registration: May 272,015).

## Background

Anthracyclines are chemotherapy agents used in the treatment for all stages of breast cancer [1]. In this therapeutic class, doxorubicin in particular is one of the most effective chemotherapeutic agents used to treat breast cancer; however, it is associated with cardiotoxicity, reduced cardiorespiratory fitness, fatigue, poor quality of life, fat gain, and muscle loss [2–4]. Anthracycline-related cardiotoxicity is of particular concern due to myocardial damage that is subclinical and that results in cardiovascular disease years later in women treated with anthracyclines [5, 6].

Exercise interventions have been utilized in breast cancer patients undergoing anthracycline-based chemotherapy to reduce some of these adverse effects [7–9]. While the previous studies showed that aerobic exercise improves cardiorespiratory fitness in breast cancer patients undergoing chemotherapy, these trials have failed to identify the optimal type, timing, and intensity of exercise intervention in these patients undergoing anthracycline-based chemotherapy [10, 11]. One exercise strategy less often used in breast cancer patients is high-intensity interval training (HIIT). HIIT includes intervals of low to high intensity aerobic exercise, and has been demonstrated to induce greater improvements in cardiorespiratory fitness than moderate continuous aerobic exercise in patients with heart failure [12] and stroke [13]. Therefore, HIIT may be a particularly effective form of exercise for breast cancer patients receiving anthracycline chemotherapy.

In other clinical exercise studies with patients with breast cancer, heart failure, and stroke, HIIT has been individually prescribed using a percentage of maximum heart rate or heart rate reserve [12, 14–17]. However, since breast cancer patients have varying resting/maximal heart rates and heart rate recovery during chemotherapy (within subject variability), utilizing heart rate to prescribe intensity does not ensure that participants will be exercising at a high intensity, which is typically defined as 80–95% of maximal heart rate [13, 18, 19]. A more valid method of prescribing intensity is to use the peak power output (PPO) generated during the exercise because it is a direct measure of the rate of external work performed [20–22]. PPO has been utilized in HIIT interventions with ranges of 90% of PPO for high intensity intervals and 10% of PPO for low intensity intervals in patients with coronary artery disease and heart failure [13, 14, 18, 23]. No studies to date have examined HIIT in breast cancer patients, using PPO to define the high and low intensity intervals.

The purpose of this pilot study was to determine whether an 8-week HIIT intervention prescribed using PPO is a feasible exercise strategy for women with early stage breast cancer receiving anthracycline. We hypothesized that more than 50% of participants randomized to HIIT would complete both an average of 70% of weekly minutes (63/90 min) and attend 70% of exercise sessions (17/24 sessions). As an exploratory aim, we sought to determine the effects of an 8-week supervised HIIT intervention on VO_2_max in breast cancer patients receiving anthracycline. We hypothesized that an 8-week HIIT intervention would maintain VO_2_max in the HIIT group compared to non-exercise control group (CON).

## Methods

### Experimental design

Detailed methods related to this study were published previously [24]. In brief, this pilot study was designed to determine the feasibility of HIIT in 30 sedentary women (less than 30 min exercise per week) undergoing potentially curative (neo)adjuvant chemotherapy for the treatment of early stage breast cancer, with doxorubicin and cyclophosphamide administered every 2 weeks for 4 cycles. All participants received white cell growth factors 24 h after chemotherapy. Participants were recruited from breast cancer clinics at the Norris Comprehensive Cancer Center and LAC+USC medical center. Once women were deemed eligible to participate in the study during their screening visit, informed consent was obtained. Participants were randomized in a 1:1 allocation to the HIIT group or CON group. Participants randomized to the HIIT group participated in an 8-week HIIT cycling intervention which included 3 times per week with each session lasting 30 min in duration. Participants randomized to the CON group were asked to maintain their current level of physical activity and were offered the HIIT intervention upon completion of the initial 8-week study period (optional HIIT participation). Outcome measures were obtained at baseline within 1 week prior to the first cycle of anthracycline (week 0), and at week 9, within 2–5 days from the last exercise session. The protocol and informed consent were approved by the institutional IRB (HS-12-00227) and registered (ClinicalTrials.gov NCT02454777).

### Eligibility

Eligibility requirements included: 1) women > 18 years of age diagnosed (stage I-III) with a first primary invasive breast cancer; 2) planned (neo)adjuvant anthracycline-based chemotherapy; 3) able to initiate the exercise intervention within 1–2 weeks of initiation of anthracycline chemotherapy; 4) less than 30 min of physical activity per week; 5) not a current smoker (no smoking in the previous 12 months); 6) willing to travel to the exercise facility at USC; 7) able to provide physician clearance to participate in the exercise intervention; 8) speak English or Spanish as a primary language. The exclusion criteria were: 1) history of chronic disease including diabetes, uncontrolled hypertension or thyroid disease; 2) weight reduction > 10% within past 6 months due to potential confounding effects on cardiorespiratory fitness; 3) metastatic disease; 4) overt CVD (myocardial infarction, stroke, angina etc.); 5) contra-indications to exercise; 6) participation in regular exercise defined as greater than 30 min exercise per week. Participants were interviewed and screened by the principal investigator (KL).

### HIIT intervention

The HIIT intervention was thrice weekly for 8 weeks. All sessions were supervised by a certified exercise trainer, performed on a stationary bike (Life Fitness 95 Elevation Series, Rosemont, IL), and took place at the USC Integrative Center for Oncology Research in Exercise. Exercise intensity was prescribed at baseline based on PPO (watts) measured with a maximal aerobic capacity fitness test performed at 60 rpm. PPO was defined as the highest power output generated during a maximal cycling test [13, 22]. Each HIIT training session included 7 times of a 1-min interval performed at 90% PPO followed by a 2-min interval performed at 10% PPO [12, 18]. Originally, exercise intensity was planned to be progressed at mid-point (week 4). However, all participants in the HIIT group did not improve VO_2_max and PPO after 4 weeks, which resulted in the same exercise intensity over the 8 weeks. Based on the previous studies, each session consisted of a 5-min warm-up performed at 10% PPO followed by the 20-min HIIT protocol (90% PPO/10% PPO), and then a 5-min cool-down (10% PPO) [13, 22]. Pedal rate or cadence (rpm), is a critical component influencing power output during cycling. Previous studies indicated that average pedal rates between 60 and 80 rpm were optimal to reach maximal aerobic capacity; we therefore set a 60 rpm pedal rate to align to previous maximal cycling protocols [25, 26]. Heart rate was recorded at the beginning and end of each 1-min, high-intensity interval as well as at the end of the 2-min recovery interval. Participants were encouraged to complete each exercise session with one full day of rest in between sessions and to complete sessions on days when they did not receive the chemotherapy infusion. If a participant preferred to perform 2–3 consecutive sessions in 1 week due to chemotherapy-related side effects (i.e., fatigue), this was documented on the exercise session form and the number of sessions with consecutive sessions in a week was counted. The purpose of this documentation was to plan for future intervention studies and to inform researchers on the preference of exercise schedules for this patient population. Participants were encouraged to make up any missed sessions in the same week, and had the ability to schedule their exercise sessions at days and times convenient for the participant alongside their chemotherapy treatment schedule.

### Feasibility measures

Adherence measures used to define feasibility were calculated for each participant by computing (1) the average weekly minutes of HIIT exercise over 8 weeks and (2) the number of sessions attended and multiplied by 100 (percentage of sessions) [27]. The overall feasibility of HIIT was assessed using both average weekly minutes of exercise completed and number of sessions attended across all participants randomized to the HIIT group. The HIIT intervention was considered feasible if more than 50% of participants completed both an average of 70% of weekly minutes (63/90 min) and attended 70% exercise sessions (17/24 sessions) [10, 28, 29]. In the event that a participant was not able to complete the entire exercise session, the number of minutes (out of 30 min) completed during each exercise session was documented. Before and after every exercise session, adverse events were monitored by checking if participant had symptoms such as nausea, vomiting, pain, and shortness of breath.

To assess if the timing of chemotherapy relative to HIIT sessions influenced the feasibility of implementing HIIT, we measured the mean number of days between each cycle of chemotherapy infusion (prescribed every 2 weeks for a total of 4 cycles of anthracycline chemotherapy) and when the participants chose to attend the HIIT sessions. By assessing the number of days during which participants were not receiving the chemotherapy infusion, we were able to assess participant preference for scheduling of exercise sessions relative to receipt of their chemotherapy. This information is particularly important because it may inform researchers and clinicians who are prescribing a structured exercise programs for these patients.

Baseline physical activity performed by each participant was assessed using the International Physical Activity Questionnaire. From this questionnaire total physical activity was calculated as follows: [Total physical activity METs-minutes/week = sum of Total (Walking + Moderate + Vigorous) METs minutes/week scores] [30, 31].

### PPO and VO_2_max

**Fig. 1:**
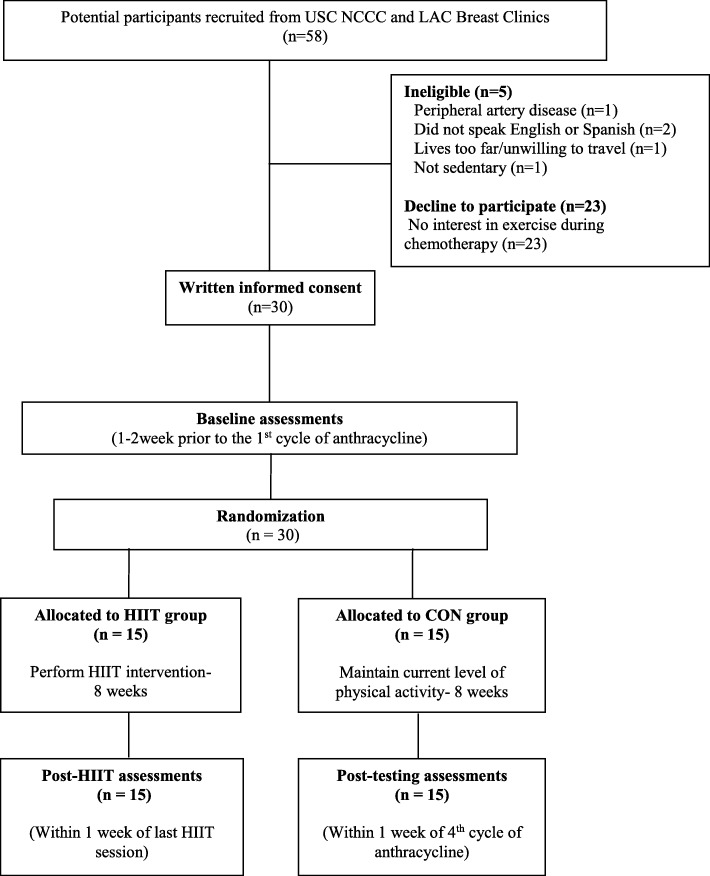
CONSORT diagram of HIIT intervention. HIIT, high intensity interval training; CON, delayed; USC, University of Southern California; NCCC, Norris Comprehensive Cancer Center; LAC, Los Angeles County

As an exploratory aim, we measured changes in VO_2_max and PPO and hypothesized that VO_2_max and PPO would maintain over 8 weeks in the HIIT group, and decrease over 8 weeks in the CON group. Prior to initiation of the HIIT intervention, all participants underwent a baseline fitness test (Week 0; Fig. 1) using a maximal cycling protocol. The specific VO_2_max protocol included a 10 W increase in workload every 60 s, starting at 40 W while maintaining 60 rpm [13]. This testing was performed with standard equipment for indirect calorimetry (Parvo Medics Inc., Salt Lake City, UT) using an incremental protocol until exhaustion on a recumbent bike. Before the fitness test was performed, participants were familiarized with the testing protocol using the same standardized verbal feedback to identify the duration of each stage, and help maintain the 60 rpm throughout the test. Participants wore a silicone face mask to collect expired air in order to determine their VO_2_ and ventilatory equivalent every 20 s. Following this testing, PPO was obtained at the last stage of maximal cycling testing.

### Statistical analysis

This study was designed as a single center pilot study, so a formal sample size calculation (i.e., to estimate numbers needed to detect a given intervention effect size) was not performed. Based on our previous work [32], we aimed to recruit roughly 2.5 participants per month in anticipation of reaching 30 participants within 1 year. Descriptive statistics were performed for all participant baseline characteristics and compared across groups to test for equivalence across the groups, using t-tests (or non-parametric Wilcoxon rank sum) for continuous variables and chi-square tests for categorical variables. The adherence measures used to define feasibility were calculated for each participant by: (1) computing the average weekly minutes of HIIT exercise (averaged over the 8 week intervention); and (2) assessing the number of sessions attended over 24 sessions and multiplied by 100 (percentage of sessions). Each of these adherence measures was averaged across all participants in HIIT group. The number and percent of HIIT participants who met both of the two feasibility criteria (average weekly minutes at least 63, and at least 17 sessions attended) was calculated. Cohen’s d effect size for the change in VO_2_max was also calculated using the difference in means from pre- and post-intervention and dividing by the pooled standard deviation. Data were analyzed using SPSS for Windows version 22 (IBM, Armonk, NY, USA).

## Results

Figure 1 depicts the study schema. All 30 participants completed their planned 8-week anthracycline-based chemotherapy in (neo)adjuvant settings. Descriptive baseline characteristics are presented in Table 1. Participants were 46.9 ± 9.8 (mean ± SD) years old, primarily Hispanic white (73%), with BMI 31.0 ± 7.5 (mean ± SD) kg/m^2^. Most participants were clinical stage II (30%) or III (63%) and received anthracycline-based chemotherapy in the neoadjuvant setting (77%). The amount of overall physical activity was not statistically different between the HIIT group (480.9 ± 85.3 METs/week) and the CON group (441.9 ± 93.2 METs/week).

**Table 1:** Baseline Participant Characteristics

	All (*N* = 30)	HIIT group (*N* = 15)	CON Group (*N* = 15)
Age, years, mean (SD)	46.9 (9.8)	49.1 (7.9)	44.7 (11.2)
Menopausal status			
Premenopausal	11 (37)	5 (33)	6 (40)
Postmenopausal	19 (63)	10 (67)	9 (60)
Body Weight, kg, mean (SD)	77.7 (18.3)	80.9 (17.7)	74.5 (18.8)
Height, cm, mean (SD)	158.4 (8.2)	156.5 (6.6)	160.3 (9.9)
BMI, kg/m^2^, mean (SD)	31.6 (7.7)	33.1 (7.6)	30.1 (7.7)
Race/ethnicity			
Non-Hispanic white	4 (13)	3 (20)	1 (6)
Hispanic white	22 (73)	11 (74)	11 (74)
African American	2 (7)	0 (0)	2 (14)
Asian/Pacific Islander	2 (7)	1 (6)	1 (6)
Disease stage			
I	2 (7)	1 (6)	1 (6)
II	9 (30)	5 (30)	4 (24)
III	19 (63)	9 (64)	10 (70)
Chemotherapy			
Neoadjuvant	23 (77)	11 (73)	12 (80)
Adjuvant	7 (23)	4 (27)	3 (20)
International physical activity questionnaire (MET min per week of moderate to vigorous intensity recreational activity), mean (SD)	462.5 (101.2)	480.9 (85.3)	441.9 (93.2)

Note. Data are presented as No. (%) unless otherwise indicated. No significant baseline differences between groups were observed (*p* > 0.05) by independent sample t tests for continuous variables and Pearson X^2^ and Fisher’s exact tests for categorical variables

Abbreviations: *BMI* body mass index, *HIIT* high intensity interval training, *DEL* delayed, *MET* metabolic equivalents, *SD* standard deviation

### Accrual and retention

Participants were recruited from August 15th 2017 to August 13th 2018. The recruitment rate was 51.7% (30/58 women) over the 12-month period. A total of 5 women screened were ineligible; reasons for ineligibility included: not willing to travel to the exercise center (*n* = 1), pre-existing chronic cardiac condition (*n* = 1), did not speak English or Spanish (*n* = 2), and high level of current physical activity participation (*n* = 1). Of 53 women screened, 23 women declined participation (43.4%). The primary reason for refusal of study participation was lack of interest in exercise during chemotherapy. All 15 enrolled in the HIIT group were retained over the 8-week HIIT intervention (100% retention).

### Feasibility (Table 2)

The mean adherence to sessions attended out of 24 sessions was 82.3%. Overall, 80% (12 of 15) of HIIT participants met both criteria; attended 19.2 ± 2.1 (mean ± SD) of 24 sessions and completed an average of 78 ± 5.1 (mean ± SD) of 90 weekly minutes of exercise over 8 weeks. Importantly, all participants in the HIIT group were able to complete prescribed 90% PPO once they initiated each HIIT session. Of the 15 participants in the HIIT group, 3 participants were not compliant (< 70% compliance). Of the remaining 12 participants, 3 participants did not schedule consecutive sessions during the intervention. Notably, 9 (80%) participants performed the intervention with consecutive HIIT sessions and reported time conflicts with their schedules as the reason for the consecutive timing. Within these 9 participants who performed consecutive sessions, a mean frequency of 2 times over the 8 week study duration occurred. This includes 6 participants with 2 times of 3 consecutive sessions (e.g. Monday, Tuesday, and Wednesday), 2 participants with 1 time of 3 consecutive sessions, and 1 participant with 4 times of consecutive sessions over the 8 weeks. Three participants were not adherent (did not meet the adherence criteria: < 70%) and reported by phone call or text message that it was due to fatigue. There were no adverse events. The mean number of days between each chemotherapy infusion and the self-selected timing of the first HIIT session each week was 1.9 ± 0.9 days (range from 1 to 5 days). The number of HIIT sessions (mean ± SD) performed during each anthracycline chemotherapy cycle was as follows: 1st cycle: 2.79 ± 0.58 sessions, 2nd cycle: 2.64 ± 0.93, 3rd cycle: 2.21 ± 1.1, and 4th cycle: 1.29 ± 1.38.

**Table 2:** Feasibility of implementing HIIT in breast cancer patients undergoing anthracycline chemotherapy

	HIIT group (*n* = 15)	CON group (*n* = 15)
Recruitment rate, %	51.7	
Retention rate, %	100	100
Adherence to HIIT, %	82.3	N/A
Minutes of HIIT per week, mean (SD)	78 ± 5.1 min	N/A
Number of HIIT sessions per each cycle of chemotherapy cycle, mean (SD)		
1st cycle	2.79 ± 0.58	N/A
2nd cycle	2.64 ± 0.93	
3rd cycle	2.21 ± 1.10	
4th cycle	1.29 ± 1.38	

Note. Data are presented as % unless otherwise indicated. No significant baseline differences between groups were observed (*p* > 0.05) Abbreviations: *BMI* body mass index, *HIIT* high intensity interval training, *CON* non-exercise control, *SD* standard deviation

### VO_2_max and PPO

**Fig. 2:**
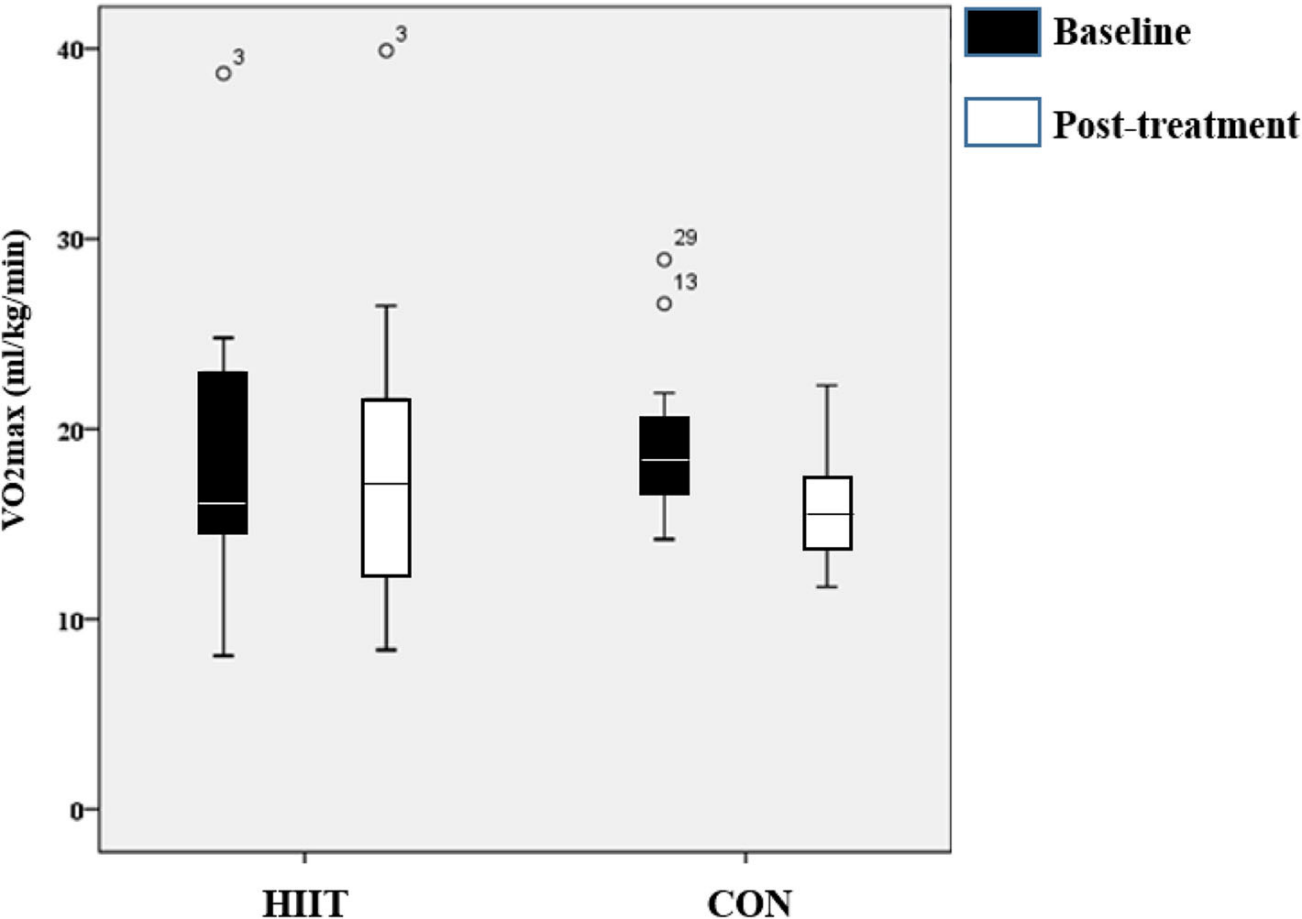
Box plot for VO2max at baseline and 8 week post-treatment in the HIIT group and the CON group

There was no statistically significant change from baseline to post-intervention in VO_2_max (19.7 ± 8.7 to 19.4 ± 6.6 ml/kg/min) in the HIIT group (*p* = 0.94) (Fig. 2). There was a significant decrease in VO_2_max (18.7 ± 7.1 to 16.1 ± 6.0 ml/kg/min) in the CON group from baseline to 8 weeks (*p* = 0.001). There was no group (HIIT vs CON) x time interaction (Pre vs Post) on VO_2_max (*p* = 0.36). There was no significant change in PPO from baseline to post-intervention in the HIIT group (122.0 ± 13.7 to 123.1 ± 20.1; *p* = 0.96). There was a significant decrease in PPO (116.4 ± 22.0 to 105.5 ± 21.1 watts) in the CON group from baseline to 8 weeks post-randomization (*p* = 0.001). The Cohen’s d effect size using the pooled standard deviation for the changes in VO_2_max (SD of change = 2.18) and PPO (SD of change = 2.54) was 1.45 and 1.19 respectively.

## Discussion

Our pilot study was the first to explore the feasibility of implementing HIIT based on PPO in breast cancer patients receiving anthracycline-based chemotherapy. We found that HIIT is indeed a feasible and safe intervention as the participants were adherent to the intervention and there were no adverse events.

We hypothesized that an 8-week HIIT exercise intervention would be a feasible exercise training approach, whereby more than 50% of participants randomized to the given condition would be able to complete both an average of 70% (63/90 min) and total prescribed weekly minutes of exercise and > 70% (17/24 sessions) of total exercise sessions with no adverse events. Based on the number of sessions attended over 24 sessions per each participant, the mean adherence to sessions attended in the HIIT group was 82.3%, and the retention rate was 100%. The mean adherence rate compares favorably with previous exercise studies in breast cancer patients undergoing chemotherapy (68–72%) [10, 11, 33]. The flexible HIIT session scheduling (6 am to 7 pm, 7 days per week or per request of the participants) and one-on-one supervision by a certified exercise trainer are likely to have contributed to the high adherence.

Although an exploratory aim, the HIIT group did not experience significant improvements in VO_2_max or PPO, while there was a significant decrease in both VO_2_max and PPO among women randomized to the CON group. Of importance, breast cancer patients experience significantly impaired cardiorespiratory fitness (~ 10%) following anthracycline-based chemotherapy [34]. Declines in cardiorespiratory fitness may not return to baseline, even years after the cessation of cancer treatment [35, 36]; thus maintaining cardiorespiratory fitness during cardio-toxic chemotherapy is a favorable finding in our study. Therefore, our study supports the use of a short-term HIIT intervention as an option to maintain cardiorespiratory fitness during anthracycline chemotherapy. This finding may be informative for a sufficiently powered trial to maintain cardiorespiratory fitness and health-related outcomes in breast cancer patients undergoing chemotherapy.

Strengths and innovations of our study include a focus on a single chemotherapy regimen, use of a novel exercise prescription using PPO, and additional assessment of feasibility measures, with the number of sessions and number of minutes attended captured. Particularly, we utilized PPO to prescribe a HIIT intervention since cancer patients during chemotherapy have unstable resting/maximal heart rate and heart rate recovery [37]; thus measuring heart rate may not be valid when prescribing a HIIT intervention. As our study cohort successfully performed HIIT interventions prescribed by PPO, PPO appears to be a useful method for prescribing/performing HIIT in breast cancer patients undergoing anthracycline chemotherapy.

Limitation include possible recruitment bias because the large percentage of patients who declined to participate in the study (39.6%) due to lack of interest in performing exercise during chemotherapy. This may have impacted on the outcomes of feasibility. In addition, we were not adequately powered to find statistically significant improvements in the changes of VO2max because this study was designed as a pilot study. Future trials are warranted to demonstrate the effects of HIIT on cardiorespiratory fitness in breast cancer patients undergoing chemotherapy.

## Conclusion

We demonstrated that HIIT prescribed by PPO for breast cancer patients receiving anthracycline-based chemotherapy is feasible. These findings await confirmation in an adequately powered study to evaluate the benefits of a HIIT intervention on health-related outcomes. Considering increasing remission and extended survival rates in breast cancer patients [38], this study provides evidence that may be used to prescribe an exercise interventions that may reduce the cardiovascular burden of anthracycline-based chemotherapy.

## Data Availability

The datasets used and/or analyzed during the current study are available from the corresponding author on reasonable request.

## Abbreviations

CON: Non-exercise control
HIIT: High intensity interval training
PPO: Peak power output
VO_2_max: Maximal oxygen uptake
